# THE USE OF MELD SCORE (MODEL FOR END-STAGE LIVER DISEASE) AND DERIVATIVES IN CARDIAC TRANSPLANTATION

**DOI:** 10.1590/0102-672020180001e1370

**Published:** 2018-07-02

**Authors:** Ana Claudia Oliveira de MORAES, Olival Cirilo Lucena da FONSECA-NETO

**Affiliations:** 1 Hospital Universitário Oswaldo Cruz, Recife, PE, Brazil

**Keywords:** Cardiac transplantation, Liver disease, Prognosis, Transplante cardíaco, Doença hepática, Prognóstico.

## Abstract

***Introduction*:**

Heart transplantation is still the best therapeutic alternative for the treatment of end-stage heart failure. The use of criteria that consider the complications associated with this procedure can guarantee a better evaluation of the recipient and prepare the team for possible unsatisfactory post-transplant results. The use of the MELD score has been expanded to evaluate cirrhotic patients undergoing various procedures, including cardiac transplantation.

***Objective*:**

To analyze the knowledge on MELD score and its derivatives to the prognosis of patients with end-stage heart failure considered for heart transplantation.

***Method*:**

Was carried out an integrative review of the publications of the last ten years in Pubmed and Lilacs databases, using the descriptors “heart transplantation”, “liver disease” and “prognosis”. From the total of 111 articles found, six were selected and composed the sample.

***Results*:**

The MELD-XI score (eXcluding INR) was the most analyzed in the studies due to the exclusion of INR, since many patients with heart failure use anticoagulants, which may alter their value. MELD and derivatives were associated with unsatisfactory results in cardiac transplantation.

***Conclusion*:**

The MELD score can be considered as a good predictor for heart transplantation; however, there are still few studies that make this correlation.

## RESUMO

*****Introdução*** ****:**

O transplante de coração ainda é a melhor alternativa terapêutica para o tratamento da insuficiência cardíaca terminal. A utilização de critérios que considerem as complicações associadas a esse procedimento pode garantir melhor avaliação do receptor e preparar a equipe para possíveis resultados insatisfatórios no pós-transplante. O uso do escore MELD vem sendo expandido para avaliar pacientes cirróticos submetidos a diversos procedimentos, incluindo o transplante cardíaco.

*****Objetivo*** ****:**

Analisar o conhecimento produzido relacionando o escore MELD e os seus derivados com o prognóstico dos pacientes com insuficiência cardíaca terminal considerados para o transplante cardíaco.

*****Método*** ****:**

Foi realizada revisão integrativa das publicações dos últimos dez anos nas bases de dados Pubmed e Lilacs, utilizando os descritores “transplante cardíaco”, “doença hepática” e “prognóstico”. Do total de 111 artigos encontrados, seis foram selecionados e compuseram a amostra.

O escore MELD-XI (eXcluding INR) foi o mais analisado nos estudos devido à exclusão do INR, já que boa parte dos pacientes com insuficiência cardíaca fazem uso de anticoagulantes, podendo alterar o seu valor. O MELD e derivados esteve associado aos resultados insatisfatórios no transplante cardíaco.

*****Conclusão*** ****:**

O escore MELD pode ser considerado como bom preditor para o transplante cardíaco; porém, ainda são poucos os estudos que fazem essa correlação.

## INTRODUCTION

Despite advances in the clinical management of heart failure (HF), transplantation is still the best therapeutic alternative for patients with terminal status, i.e. those with functional class III or IV and who present major limitations and high mortality in one year. The selection of these candidates for transplantation depends on careful, clinical and functional evaluation, with the objective of reducing unfavorable outcomes^4^ ^,^ ^5^ ^,^ ^18^ ^,^ ^19^ ^,^ ^21^.

Heart Failure Survival Score (HFSS) and Seattle Heart Failure Model (SHFM) were established to assess survival in patients with HF at the outpatient level, and may guide the need for transplantation. However, these scores are not applied in hospitalized patients and/or with other comorbidities, such as renal failure, liver dysfunction, cancer, among others, which may be present in patients with terminal HF, leading to worsening prognosis. Despite the existence of other parameters to evaluate the risks of heart transplantation, such as the cardiopulmonary test, its predictive capacity is not so effective when there is an association of dysfunction of other organs^4^ ^,^ ^6^ ^,^ ^16^.

Congestive hepatopathy or cardiac cirrhosis are well-known consequences of terminal HF as well as altered renal function, which occurs due to increased congestion associated with ventricular dysfunction and decreased perfusion. Recent studies have investigated the use of the Model for End-Stage Liver Disease (MELD) score in patients with terminal HF, considering their potential predictor of outcomes and their ability to assess liver and renal function simultaneously.

The MELD score was described to predict the three-month survival rate of patients submitted to transjugular intrahepatic portosystemic shunt and validated as a measure of probability of mortality in patients candidates for liver transplantation^11^ ^,^ ^24^ ^,^ ^31^. Since then, it has been adopted as a criterion in the allocation of liver for transplantation until the present day. A logarithmic calculation with serum creatinine, bilirubin and International Normalized Ratio (INR) results in the MELD score for patients who are candidates for liver transplantation over 12 years^2^ ^,^ ^12^ ^,^ ^17^.

The use of this score has been expanded to predict mortality risk in cirrhotic patients submitted to orthopedic, digestive and cardiovascular surgery and, more recently, has been associated with cardiac liver disease candidates for heart transplantation^6^ ^,^ ^27^ ^,^ ^28^ ^,^ ^30^. However, the prognostic value of the MELD score may be limited when using anticoagulants, as is the case with most HF patients. And, alternatively, the MELD-XI (eXcluding INR) score, derived from MELD, has been more appropriately used in these situations, since it excludes INR since there is a change in its value due to the use of this medication, improving its prognostic power. High values ​​of this score have been related to unsatisfactory results not only in liver transplantation, but also in cases of terminal HF that were considered for heart transplantation^6^ ^,^ ^15^ ^,^ ^20^ ^,^ ^27^ ^,^ ^30^.

According to the above, this study aimed to analyze the knowledge produced on the use of MELD score and derivatives related to cardiac transplantation, considering the following question as guideline: What is the relationship of the MELD score and derivatives with the prognosis of the patients with terminal HF evaluated for heart transplantation?

## METHOD

An integrative review was made considering articles published in the last ten years and indexed to Pubmed and Latin American Literature in Health Sciences (Lilacs). For the survey of the articles, the controlled descriptors of the Virtual Health Library were used through Mesh (Medical SubjectHeadings) consisting of “heart transplantation” and “liver diseases” and “prognosis”. The initial research revealed 53 articles in Pubmed and 58 in Lilacs, with complete texts in English and Spanish (Lilacs) and, through the reading of titles and abstracts, six articles from each base were selected, three being excluded by repetition, remaining nine articles.

In these articles an instrument of evaluation of the methodological rigor of the selected articles adapted from Critical Appraisal Skills Program (CASP)^22^ was applied containing questions about the clarity of the objective, methodological suitability, theoretical-methodological procedures, sample selection, researcher and researched relationship, ethical aspects, rigor and fundamentals of data analysis, statement of results and importance of the research.

The evaluated items received a point, and from the sum of the scores the result was obtained. Those that were scored from six to ten were considered with good methodological quality and reduced bias (level A)^22^, remaining in the sample.

**FIGURE 1:**
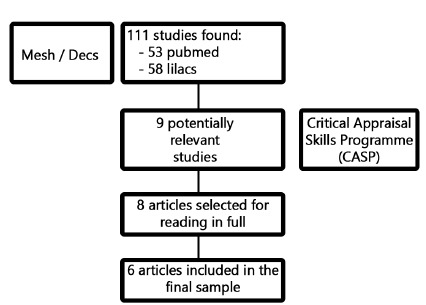
Flow diagram representative of the article selection process

Based on this instrument, one article was deleted and the other (8) read in full. Out of this total, six answered the guiding question and defined the final sample of the present review (Figure 1).

## RESULTS

**FIGURE 2:**
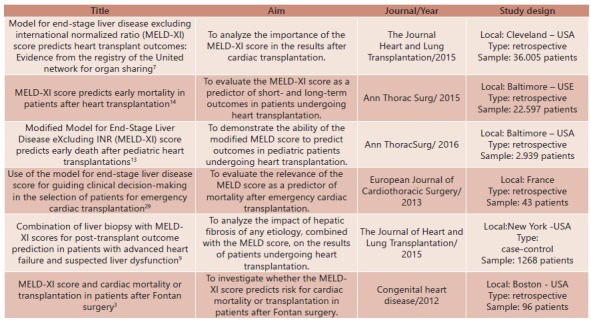
Selected studies in the integrative review using the MELD score and derivatives in cardiac transplantation

Most were retrospective cohort studies published in the year 2015, predominantly conducted in the United States and only one in France. Selected articles and publications were characterized and presented in Figure 2.

There was a significant difference between sample sizes, ranging from 4329 to 36.0057 patients. Those who underwent heart transplantation alone were considered. Prevalence of male and middle age (45-55 years) prevailed in the samples, except in the studies that evaluated the application of the MELD-XI score in the pediatric transplantation^13^ and after the Fontan operation^3^.

The MELD and MELD-XI scores were obtained from the following logarithmic formulas, respectively: 3.78xLn (total bilirubin) + 11.2xLn (INR) + 9.57xLn (creatinine) + 6.4317 and 5.11xLn (total bilirubin) + 11.76xLn Creatinine) + 9.4415, where total bilirubin and creatinine were equal to 1 if the laboratory value was &lt;1. Another MELD derived score, modMELD, was used in only one study^9^, and its calculation followed the same MELD formula, replacing INR with albumin.

A considerable number of patients with HF and, especially those who require ventricular assist devices, use anticoagulants which lead to an increase in the value of INR. Because of this issue, the majority of studies used the MELD-XI score to assess cardiac transplant outcomes.

It was not possible to obtain consensus between the cut points established for the used score. The MELD-XI score range was between 10,677, the lowest cut-off point, and 183, the highest. This high score (considering the various cut points) was associated with worse cardiac transplant results, showing a strong relation with morbidity and mortality, especially in the first postoperative year^7^ ^,^ ^9^ ^,^ ^13^ ^,^ ^14^ ^,^ ^29^.

In addition, there was a correlation between this score and the post-transplant prevalence of infections, rejection, cerebral vascular accidents, longer mechanical ventilation, prolonged hospitalization, and renal failure requiring dialysis^7^ ^,^ ^9^ ^,^ ^13^ ^,^ ^14^ ^,^ ^29^.

Vanhuyse et al^29^ used only MELD in their study to predict the occurrence of mortality in patients who underwent emergency cardiac transplantation and confirmed the predictive capacity of this score. Nevertheless, in cases of advanced HF, the MELD-XI score seems to have a higher prognostic value in relation to MELD.

In the Farret al^9^ study, the association of MELD-XI with liver biopsy in patients with terminal HF and suspected liver dysfunction was performed. From this study, a hepatic risk score was created using the degrees of fibrosis, based on a scale from 0 to 4 according to the biopsy, in addition to MELD-XI. Thus, this new score is obtained from the following calculation: (fibrosis + 1) x (MELD-XI). One-year survival was 95.5% in patients with hepatic risk score &lt;45, but 42.9% in those with a score ≥45.

Assenza et al^3^ evaluated the increased risk of cardiac mortality or for transplantation in patients after Fontan operation associated with the MELD-XI score. The results were similar when it came to the good pre-surgical predictive capacity of this score. The higher the value, the greater the chances of sudden death or need for heart transplantation. In addition, although the MELD-XI score was not applied in pediatric cardiac transplantation, its predictive power of morbidity and mortality after surgery^13^ was also evidenced.

## DISCUSSION

Significant hepatic dysfunction is reported in several studies as being frequently found in patients with advanced HF, and may manifest as congestive hepatopathy and cardiac cirrhosis, or in other ways. Hepatic function markers (aspartate aminotransferase, alanine aminotransferase, bilirubin, alkaline phosphatase, etc.), although showing significant improvement after a few months of heart transplantation, were also related to unsatisfactory results, since the reversibility in this case will depend on the degree of hepatic parenchyma impairment^6^ ^,^ ^8^.

On the other hand, despite the increase in transplantation, the disproportion between the number of recipients and organ donors is still considerable. This situation increases the waiting time for the organ, contributes to the worsening of the disease and the indication of circulatory assistance devices, which can lead to an increase in pre-transplant survival and serve as a bridge for transplantation, but also influences post-transplantation outcomes. In this way, the most appropriate selection of the receptor is of great importance for successful results^1^ ^,^ ^18^ ^,^ ^23^ ^,^ ^26^.

The MELD score, initially used in liver allocation for transplantation, has been shown to be applicable in other situations. Chokshi et al^6^ evaluated patients with cardiac hepatopathy who underwent heart transplantation using parameters of hepatic dysfunction using the MELD and modMELD scores. The high value of these scores was associated with complications and worse prognosis after cardiac transplantation.

Considering the frequent use of anticoagulant therapy, the MELD-XI score was predominantly evaluated in selected studies, showing good accuracy as an independent predictor of transplant mortality and/or among patients requiring a ventricular assist device. The value above the cutoff points of this pre-transplant score was related to the worse prognosis after the operation, confirming the importance of better understanding the hepatic and renal dysfunctions and their influence on survival^1^ ^,^ ^3^ ^,^ ^7^ ^,^ ^9^ ^,^ ^13^ ^,^ ^14^ ^,^ ^30^.

Despite this, Grimm et al. observed that creatinine and bilirubin compared to the MELD-XI score were higher in terms of the predictive power of mortality at one year. Several studies have shown reduced survival in adult patients when evaluated through this score. However, in pediatric transplantation, the use of this score is still limited^2^ ^,^ ^3^ ^,^ ^13^ ^,^ ^15^ ^,^ ^19^.

The association of the MELD-XI score with the worst prognosis in cardiac transplantation was significant; however, the parameters used to obtain this score were dynamic, and could be increased or decreased according to HF therapy, support mechanisms or progression of the condition, important to update the calculation. In addition, in cases where there is evidence of liver fibrosis or cirrhosis, conducting further investigation, such as biopsy, contributes to diagnosis and prognosis. Although the biopsy is still the most accessible method in these situations, other techniques have been developed and with the ability to accurately and safely assess hepatic impairment and non-invasively, such as transient hepatic elastography^1^ ^,^ ^8^ ^,^ ^10^.

In cases where there is irreversible liver damage, double heart-liver transplantation may be considered. Despite the greater risk and probable decrease of survival, when indicated at the best moment of the patient and performed, favorable results can be obtained. Schaffer et al., showed that double transplantation (heart-liver) was related to better survival for patients with heart and liver failure^8^ ^,^ ^9^ ^,^ ^25^.

## CONCLUSION

The studies confirmed the predictive power of the MELD score and derivatives, showing its applicability in cardiology. The use of this tool can contribute to the evaluation of the severity of the patients with HF who are candidates for transplantation earlier and in the definition of priority, as in liver transplantation. However, although the high MELD-XI score (more specifically) is related to the worse prognosis, teams should not be discouraged about performing the procedure, but should prepare themselves for better management of possible complications. And, despite the favorable results to the use of this score, there are still few and recent studies relating it to heart transplantation or to other clinical practices besides liver transplantation.

## References

[1] Abe S, Yoshihisa A, Takiguchi M, Shimizu T, Nakamura Y, Yamauchi H (2014). Liver Dysfunction Assessed by Model for End-Stage Liver Disease Excluding INR (MELD-XI) Scoring System Predicts Adverse Prognosis in Heart Failure. PLoS One Jun.

[2] Andraus W, Haddad L, Rocha-Santos V, D'Albuquerque LAC (2013). Análise dos sistemas de alocação de órgãos para transplantes do aparelho digestivo no Brasil. Medicina (RibeirãoPreto).

[3] Assenza GE, Graham DA, Landzberg MJ, Valente AM, Singh MN, Bashir A (2013). MELD-XI score and cardiac mortality or transplantation in patients after Fontan surgery. Heart Apr.

[4] Bacal F, Souza-Neto JD, Fiorelli AI, Mejia J, Marcondes-Braga FG, Mangini S (2009). II Diretriz Brasileira de Transplante Cardíaco. Arq Bras Cardiol.

[5] Bocchi EA, Marcondes-Braga FG, Ayub-Ferreira SM, Rohde LE, Oliveira WA, Almeida DR (2009). Sociedade Brasileira de Cardiologia. III Diretriz Brasileira de Insuficiência Cardíaca Crônica. Arq Bras Cardiol.

[6] Chokshi A, Cheema FH, Schaefle KJ, Jiang J, Collado E, Shahzad K (2012). Hepatic dysfunction and survival after orthotopic heart transplantations Application of the MELD scoring system for outcome prediction. J Heart Lung Transplant.

[7] Deo SV, Al-Kindi SG, Altarabsheh SE, Hang D, Kumar S, Ginwalla MB (2016). Model for end-stage liver disease excluding international normalized ratio (MELD-XI) score predicts heart transplant outcomes Evidence from the registry of the United Network for Organ Sharing. J Heart Lung Transplant.

[8] Dichtl W, Vogel W, Dunst KM, Grander W, Alber HF, Frick M (2005). Cardiac hepatopathy before and after heart transplantation. Transpl Int.

[9] Farr M, Mitchell J, Lippel M, Kato TS, Jin Z, Ippolito P (2015). Combination of liver biopsy with MELD-XI scores for post-transplant outcome prediction in patients with advanced heart failure and suspected liver dysfunction. J Heart Lung Transplant.

[10] Fraquelli M, Pozzi R (2012). The accuracy of noninvasive methods in the prediction of clinically relevant outcomes in patients with chronic liver disease. Expert Rev Gastroenterol Hepatol.

[11] Freitas ACT, Shiguihara RS, Monteiro RT, Pazeto TL, Coelho JCU (2016). Comparative study on Liver Transplantation with and without Hepatocellular Carcinoma with cirrhosis: analysis of MELD, waiting time and survival. ABCD, Arq Bras Cir Dig.

[12] Freitas AC, Itikawa WM, Kurogi AS, Stadnik LG, Parolin MB, Coelho JC (2010). The impact of the model for end-stage liver disease (MELD) on liver transplantation in one center in Brazil. Arq Gastroenterol.

[13] Grimm JC, Magruder JT, Do N, Spinner JA, Dungan SP, Kilic A (2016). Modified Model for End-Stage Liver Disease eXcluding INR (MELD-XI) Score Predicts Early Death After Pediatric Heart Transplantation. Ann Thorac Surg.

[14] Grimm JC, Shah AS, Magruder JT, Kilic A, Valero 3rd V, Dungan SP (2015). MELD-XI Score Predicts Early Mortality in Patients After Heart Transplantation. Ann Thorac Surg Nov.

[15] Heuman DM, Mihas AA, Habib A, Gilles HS, Stravitz RT, Sanyal AJ (2007). MELD-XI a Rational Approach to "Sickest First" Liver Transplantation in Cirrhotic Patients Requiring Anticoagulant Therapy. Liver Transpl.

[16] Inohara T, Kohsaka S, Shiraishi Y, Goda A, Sawano M, Yagawa M (2014). Prognostic impact of renal and hepatic dysfunction based on the MELD-XI score in patients with acute heart failure. Int J Cardiol.

[17] Kamath PS, Wiesner RH, Malinchoc M, Kremers W, Therneau TM, Kosberg CL, D'Amico G, Dickson ER, Kim WR (2001). Predict survival in patients with end-stage liver disease. Hepatology.

[18] Mangini S, Alves BR, Silvestre OM, Pires PV, Pires LJT, Curiati MNC, Bacal F (2015). Transplante cardíaco revisão. Einstein.

[19] Ministério da Saúde Portaria nº 2600, de 21 de outubro de 2009. Aprova o regulamento técnico do Sistema Nacional de Transplantes.

[20] Murata M, Kato TS, Kuwaki K, Yamamoto T, Dohi S, Amano A (2016). Preoperative hepatic dysfunction could predict postoperative mortality and morbidity in patients undergoing cardiac surgery Utilization of the MELD scoring system. Int J Cardiol.

[21] Ponikowski P, Voors AA, Anker SD, Bueno H, Cleland JGF, Coats AJS (2016). 2016 ESC Guidelines for the diagnosis and treatment of acute and chronic heart failure. European Heart Journal.

[22] Public Health Resource Unit, The University of Kent, Critical Appraisal of the Journal Literature (2006). Critical Appraisal Skills Programme (CASP) - Evaluation tool for quantitative studies.

[23] Registro Brasileiro de Transplantes Associação Brasileira para Transplante de Órgãos (ABTO). Dados numéricos da doação de órgãos e transplantes realizados por estado e instituição no período: janeiro/setembro 2016.

[24] Sá GPD, Vicentine FPP, Salzedas-Netto AA, Matos CAL, Romero LR, Tejada DFP (2016). Liver Transplantation for Carcinoma Hepatocellular in São Paulo: 414 cases by the Milan/Brazil criteria. ABCD, Arq Bras Cir Dig Dec.

[25] Schaffer JM, Chiu P, Singh SK, Oyer PE, Reitz BA, Mallidi HR (2014). Combined heart-liver transplantation in the MELD era do waitlisted patients require exception status?. Am J Transplant.

[26] Szygula-Jurkiewicz B, Zakliczynski M, Szczurek W, Nadziakiewicz P, Gasior M, Zembala M (2016). Predictive Value of the Model for End-Stage Liver Disease Score Excluding International Normalized Ratio One Year After Orthotopic Heart Transplantation. Trasplant Proc.

[27] Szygula-Jurkiewicz B, Nadziakiewicz P, Zakliczynski M, Szczurek W, Chraponski J, Zembala M (2016). Predictive Value of Hepatic and Renal Dysfunction Based on the Models for End-Stage Liver Disease in Patients With Heart Failure Evaluated for Heart Transplant. Transplantat Proc.

[28] Teh SH, Nagorney DM, Stevens SR, Offord KP, Therneau TM, Plevak DJ (2007). Risk factors for mortality after surgery in patients with cirrhosis. Gastroenterology Apr.

[29] Vanhuyse F, Maureira P, Mattei MF, Laurent N, Folliguet T, Villemot JP (2013). Use of the model for end-stage liver disease score for guiding clinical decision-making in the selection of patients for emergency cardiac transplantation. Eur J Cardiothorac Surg.

[30] Yang JA, Kato TS, Shulman BP, Takayama H, Farr M, Jorde UP (2012). Liver dysfunction as a predictor of outcomes in patients with advanced heart failure requiring ventricular assist device support Use of the Model of End-stage Liver Disease (MELD) and MELD eXcluding INR (MELD-XI) scoring system. J Heart Lung Transplant.

[31] Zanchet MV, Silva LLG, Matias JEF, Coelho JCU (2016). Post-reperfusion liver biopsy and its value in predicting mortality and graft dysfunction after Liver Transplantation. ABCD, Arq Bras Cir Dig.

